# Chronic Consumption of Ghee (Clarified Butter) and Coconut Oil, but Not Palmolein, Induces Microvesicular Steatosis, Insulin Resistance, and Inflammation in Mice Fed a High-Fat Diet

**DOI:** 10.3390/nu18213642

**Published:** 2026-10-31

**Authors:** Suryanarayana Palla, Suryam Reddy Kona, Surekha Venkata Mullapudi, Princitai Tejram Shahare, Saikanth Varma, Archana Molangiri, Sanjay Basak, Ahamed Ibrahim

**Affiliations:** 1 Lipid Chemistry Division, ICMR-National Institute of Nutrition, Hyderabad 500007, India; 2 Department of Pathology, ICMR-National Institute of Nutrition, Hyderabad 500007, India; 3 Molecular Biology Division, ICMR-National Institute of Nutrition, Hyderabad 500007, India

**Keywords:** MASLD, clarified butter, visceral adiposity, microvesicular steatosis, palmolein, coconut oil

## Abstract

**Background/Objectives:** A high-calorie diet contributes to the development of metabolic dysfunction-associated steatotic liver disease (MASLD). Excessive saturated fat intake can lead to metabolic dysfunction, depending on the sources, quality, and duration of the oils and fats consumed. Palmolein, ghee, and coconut oil are common dietary fats that differ in saturated fatty acid (SFA) content. However, the impact of these fats on liver steatosis remains unclear. **Methods:** Forty adult male C57BL/6 mice were divided into five groups (*n* = 8/group): chow diet (5% *w*/*w* peanut oil) and high-fat diets (HFDs) enriched with peanut, palmolein, ghee, and coconut oil (all 25% *w*/*w*). Peanut oil, which is a traditional cooking oil, was used as a reference. All the HFD groups were isocaloric and received 58.4% of their energy from fat. **Results:** After 6 months, bodyweight gain and fasting glucose were elevated in all HFD-fed groups (*p* < 0.05), except in the peanut oil-fed group. The ghee-fed group showed the highest feed efficiency, visceral adiposity, insulin resistance, plasma triglycerides (TGs), leptin, liver TGs, fatty liver score, interleukin 6 (IL-6) expression, and TBARS levels (*p* < 0.05). Unlike the ghee-fed group, the coconut oil-fed group exhibited insulin resistance, hepatic lipid accumulation, and IL-6 expression (*p* < 0.05), independent of visceral adiposity and oxidative stress. The palmolein oil-fed groups showed minimal changes in fatty liver score (*p* > 0.05) compared with the ghee- and coconut oil-fed groups. **Conclusions:** In the HFD setup, chronic intake of ghee- and coconut oil-enriched diets induces microvesicular steatosis and inflammation in the liver compared with the peanut and palmolein diets by triggering insulin resistance and dyslipidemia.

## 1. Introduction

Although MASLD is multifactorial, nutritional factors, including excess calorie intake, contribute to its development [1]. Excessive TGs, a hallmark of MASLD, is closely associated with obesity, insulin resistance, and abnormal lipid metabolism [2]. Dietary fat, both the amount and composition, may play a pivotal role in the development of MASLD since the quality of dietary fat appears to influence intrahepatic TG accumulation [3]. Evidence suggests that a higher SFA intake leads to greater visceral and hepatic fat accumulation than unsaturated fatty acid (UFA) intake [4]. Increased SFA intake was found to be more metabolically harmful for the human liver than simple sugar or UFAs due to its greater induction of intrahepatic TG content, insulin resistance, and harmful ceramides [5].

Dietary SFAs showed a greater stimulatory effect on weight gain and hepatic lipid accumulation than UFAs due to the overflow of fat to the distal intestine, which induced changes in the gut microbiota and host metabolism [6]. An evaluation of twelve commonly consumed dietary fats in mice showed that fat source differentially influenced body weight, circulating TGs, and gut microbiota composition, indicating that different dietary fats elicit distinct metabolic effects, in part through their differential impact on the composition and functional activity of the host gut microbiota [7]. Moreover, dietary fatty acid composition interacts with the gut microbiota to influence hepatic steatosis and host metabolism [8]. Furthermore, studies comparing different HFDs have reported variable effects on metabolic outcomes. Mice fed an HFD containing lard, soybean oil, and milk fat for 12 weeks exhibited comparable weight gain and hepatic steatosis. In contrast, milk fat increased serum cholesterol and advanced glycation end products more than lard or soybean oil [9]. Similarly, feeding an HFD containing lard, palmolein, or interesterified palmolein resulted in increased bodyweight gain, fasting glucose, homeostasis model assessment of insulin resistance (HOMA-IR), and hepatic steatosis in all HFD groups. Still, the magnitude of these effects differed by fat type [10].

Tropical oils such as coconut oil and palmolein oil, which contain high levels of SFAs, are integral components of Indian cuisine. Moreover, in recent years, the food industry has used palm oil fractions such as palmolein, palm stearin, and palm kernel oils extensively as an alternative to partially hydrogenated vegetable oils after trans-fat regulations were implemented in India. In addition to tropical oils, ghee (clarified butter), a specialized milk fat derived from butter by removing water and nonfat solids through steeping and skimming, also contains high levels of SFAs. Because of its high smoke point, ghee is often used as a frying medium, in the preparation of sweets, and as a spread or topping for cooked food items. Current dietary guidelines recommend limiting SFAs to <10% of total calories and replacing them with UFAs to prevent diet-related metabolic disorders [11]. A recent observational analysis of Indian adults showed that each two-percentage-point increase in energy intake from SFAs was associated with ~ 6% higher odds of elevated low-density lipoprotein (LDL) cholesterol, suggesting that predominant cooking-fat choice may be a modifiable dietary determinant of metabolic risk [12]. Palmolein, ghee, and coconut oil are commonly used dietary fat sources that differ markedly in their SFA content and fatty acid composition. Short-term studies have reported the diverse metabolic effects of peanut [13], palmolein oil [14,15,16], ghee [7,17,18], and coconut oil [19,20] on hepatic lipid deposition and associated factors.

Despite available data, the long-term impact of an HFD enriched with palmolein, ghee, and coconut oil on hepatic steatosis remains limited. Therefore, the study evaluated the effects of these dietary fats, each incorporated at 25% (*w*/*w*) in an HFD for 6 months, on hepatic fat deposition and relevant metabolic parameters in C57BL/6 mice. Peanut oil, a traditional cooking oil in India, was used as the reference oil. All HFDs were isocaloric but differed in SFA, MUFA, and PUFA contents. Because fatty acid turnover is slow, longer-term comparative measurements of increasing levels of SFA-enriched dietary oils and fats can provide early metabolic insights into their contribution to MASLD development.

## 2. Materials and Methods

### 2.1. Diet

The chow diet was prepared as per AIN-93M recommendations and is presented in Table 1.

Peanut oil, buffalo ghee, and palmolein oil were procured from the local market, while cold-pressed coconut oil was obtained from local manufacturers. The fatty acid composition of the dietary fat sources is presented in Table A1. HFDs were prepared by thoroughly mixing the respective oils/fat with chow diet (75% chow diet without oil and 25% respective oils). The chow diet provided 303 kcal/100 g diet, with 25% of calories derived from fat, whereas HFD provided 428 kcal/100 g diet, with 58.4% of calories derived from oil/fat, and all the HFDs were isocaloric. The contents of total SFAs, MUFAs, and PUFAs in the dietary oils and fats were analyzed by gas chromatography, as described later. The fatty acid composition of the experimental diets is presented in Table 2.

### 2.2. Animal Experiment

The animal experiment was conducted as per the guidance of the committee for the control and supervision of experiments on animals, Government of India, and complied with the ARRIVE guidelines. The Institutional Animal Ethical Committee of ICMR-National Institute of Nutrition (NIN), Hyderabad, India, approved the research protocol (NIN/IAEC/2022-II/007). Three-month-old male C57BL/6 mice (*n* = 40) were obtained from the animal house facility of ICMR-NIN. Mice were housed individually in wire mesh-bottomed polypropylene cages under controlled environmental conditions (22 ± 2 °C, 12 h light/12 h dark cycle) with free access to food and water. Mice were acclimatized on a chow diet (AIN-93M) for 7 days. After that, mice were randomly divided based on body weight into five groups (*n* = 8 per group) and fed one of the following diets for 6 months: (1) Chow diet containing 5% peanut oil, (2) HFD containing 25% peanut oil, (3) HFD containing 25% palmolein oil, (4) HFD containing 25% ghee, and (5) HFD containing 25% coconut oil. Daily food intake was recorded, and body weights were measured once every ten days. All animals were sacrificed at the end of the six-month period.

### 2.3. Blood Collection and Biochemical Assays in Plasma

At the end of the metabolic trial, mice were fasted for 6 h prior to blood collection. Blood was collected from the retro-orbital plexus and stored in EDTA-coated vials. Plasma was separated and stored (−80 °C) for subsequent analysis. Plasma glucose (Biosystems, Barcelona, Spain) and alanine aminotransferase (ALT) activity (Span Diagnostics Ltd., India) were estimated on the same day using commercial kit methods as per their instructions. Plasma insulin levels were measured using an ELISA kit (Mercodia AB, Sweden). Insulin resistance was assessed using the HOMA-IR index, calculated as: HOMA-IR = Fasting glucose (mmol/L) × Fasting insulin (mU/L)/22.5. Plasma leptin levels were measured using an ELISA kit (Elabscience, USA). Total cholesterol (TC), TGs, and high-density lipoprotein cholesterol (HDL-C) were estimated in plasma using commercial kits (Biosystems, Spain).

### 2.4. Termination and Organ and Tissue Collection

After blood collection, mice were euthanized by CO_2_ asphyxiation. Liver, retroperitoneal (RP) fat, and epididymal (EP) fat tissues were excised, rinsed with saline, weighed, snap-frozen in liquid nitrogen, and stored at −80 °C for further analyses. A portion of liver tissue was preserved in RNA later (Sigma-Aldrich, India) for transcriptome analysis, while another portion was fixed in 10% neutral buffered formalin for histological examination. A portion of the liver tissue was also snap-frozen in liquid nitrogen for subsequent protein expression analysis. Adiposity index was calculated as the sum of epididymal and retroperitoneal adipose tissues (g)/body weight (g) × 100 [21].

### 2.5. Determination of TGs and TC in the Liver

Briefly, 100 mg of liver tissue was homogenized in 5% aqueous Nonidet P-40 (NP-40; Sigma, India) solution. The homogenate was gradually heated to 80 °C in a water bath until the solution became cloudy, cooled to room temperature, and reheated once more to ensure complete lipid solubilization, then centrifuged at 3000 rpm for 20 min, as described previously [22]. The TGs and TC were measured in the supernatant using commercial assay kits as per the supplier’s instructions (Biosystems, Barcelona, Spain).

### 2.6. Fatty Acid Compositional Analysis of the Diets

Dietary lipid extraction was performed by adding 250 µg of heptadecanoic acid (C17:0; mg/mL) as an internal standard and 20 mL of chloroform: methanol (2:1, *v*/*v*) containing butylated hydroxytoluene (BHT; 10 mg/L) to 200 mg of diet sample. Samples were mixed thoroughly, flushed with nitrogen, and incubated overnight. After filtration, 3 mL of water was added to the filtrate, vortexed, and allowed to stand for 30 min. The lower chloroform layer was collected. The aqueous phase was re-extracted twice with chloroform: methanol (2:1, *v*/*v*), and the pooled chloroform extracts were evaporated to dryness under nitrogen. The dried lipid extract was saponified, followed by acidification and methylation. The final extract was stored at −20 °C until fatty acid analysis by gas chromatography (PerkinElmer Clarus 680, USA).

### 2.7. Estimation of Hepatic Lipids by Fatty Acid Composition

Total lipids from liver tissue were extracted using the Folch method. A 10% liver homogenate (500 µL) was mixed with 5 mL of chloroform: methanol (2:1, *v*/*v*), vortexed vigorously, filtered, and reextracted with an additional 3 mL of solvent. The pooled extract was washed with one-fifth volume of 0.22% KCl and centrifuged at 2000 rpm for 2 min. The lower organic phase was collected and evaporated under nitrogen. The dried extract was spiked with a known quantity of heptadecanoic acid (C17) internal standard and methylated using 2% sulfuric acid in methanol containing BHT (10 mg/L) at 70 °C for 4 h. After cooling, fatty acid methyl esters were extracted with petroleum ether, washed with 0.2 M sodium bicarbonate, dried over anhydrous sodium sulfate, and stored at −20 °C until analysis. Fatty acid composition was analyzed using gas chromatography (PerkinElmer Clarus 680, USA) equipped with a flame ionization detector and an SP-2300 fused silica capillary column (30 m × 0.25 mm × 0.2 μm; Supelco, USA). Fatty acids were identified by comparison with authentic standards (Nuchek, USA) based on retention times.

### 2.8. Histology and MASLD Activity Score

Formalin-fixed liver tissues were embedded in paraffin blocks, sectioned at 5 µm thickness, and mounted on glass slides. Sections were stained with hematoxylin and eosin (H&E) and examined under a light microscope for hepatic steatosis and lobular inflammation. The analysis included 40 mice, with a minimum of five non-overlapping microscopic fields assessed per mouse, for a total of at least 200 fields. Histopathological assessment was performed under blinded conditions by systematically examining entire tissue sections and quantitatively evaluating multiple fields per animal. Relevant alterations, including microvesicular steatosis and hepatocyte ballooning, were documented using standardized histopathology forms. Representative photomicrographs were captured from the sections studied simultaneously. The severity of MASLD was assessed using the MASLD activity score (MAS), as described before [23]. MAS was calculated based on steatosis (0–3), lobular inflammation (0–3), and hepatocyte ballooning (0–2) in a blinded manner. An MAS score > 4 was considered indicative of MASH (metabolic dysfunction-associated steatohepatitis).

### 2.9. Lipid Peroxidation and Antioxidant Enzymes in the Liver

Lipid peroxidation levels in the liver were estimated by measuring malondialdehyde (MDA) using the thiobarbituric acid reactive substances (TBARS) assay [24]. Catalase, glutathione peroxidase, and reduced glutathione in the liver supernatant were measured using assays as mentioned before [22].

### 2.10. Gene Expression Analysis by Real-Time qPCR

Total RNA was isolated from liver tissue using TRI reagent (Cat#T9424, Sigma, India) according to the manufacturer’s protocol. Briefly, 50 mg of liver tissue was homogenized in TRI reagent (Cat#T9424, Sigma, India) using 2 mm zirconia beads in a bead beater (Mini Bead Beater, BioSpec, USA). Phase separation was achieved by the addition of chloroform, and RNA was precipitated with isopropanol, followed by dissolution in nuclease-free water as described previously [25]. Total RNA was purified using DNase 1 (Cat#AMPD1, Sigma, India) to remove any traces of contaminating DNA. The quality and quantity of total RNA were assessed by a spectrophotometer (Multiskan Skyhigh, Thermo Scientific, USA). cDNA was synthesized using a Bio-Rad iScript cDNA synthesis kit (Cat#1708891, Bio-Rad, USA). Real-time qPCR was performed in a Bio-Rad CFX 96 system (Bio-Rad, USA) using KiCqStart SYBR green primers (Table A2) and TB green pre-mix Ex-Taq II (Cat#RR208, Takara, Japan). The relative mRNA expression was quantified by the ddCt method, and β-actin was used as an endogenous reference gene. Values were obtained from repeated independent experiments (*n* = 6 per experimental group), with each sample analyzed in duplicate.

### 2.11. Immunoblotting

Hepatic expression of the pro-inflammatory cytokine IL-6 protein was determined by Western blotting. Briefly, approximately 100 mg of liver tissue was finely pulverized under liquid nitrogen and homogenized in RIPA lysis buffer (pH 7.5) supplemented with protease inhibitors. The homogenates were centrifuged at 12,000× *g* at 4 °C for 20 min, and the resulting supernatants were collected for protein analysis. Approximately 20 µg of total protein from each sample was separated by electrophoresis on a 12% polyacrylamide gel and subsequently transferred onto a nitrocellulose membrane. Following blocking, the membrane was incubated overnight at 4 °C with a rabbit anti-IL-6 primary antibody (#P620, Thermo Fisher Scientific, USA) at a dilution of 1:2500. The membrane was subsequently incubated with an HRP-conjugated anti-rabbit secondary antibody at a dilution of 1:5000. Immunoreactive bands were visualized using an enhanced chemiluminescence substrate (#1705061, Bio-Rad, USA) and captured using a G-BOX gel imaging system (Syngene, UK). Band intensities were quantified by densitometric analysis using ImageJ version 1.50i (NIH, USA). IL-6 protein expression was normalized to the corresponding β-actin loading control, and the normalized data were subjected to statistical analysis as described in the respective figure legend.

### 2.12. Statistical Analysis

All data are expressed as mean ± standard error of the mean (SEM). Statistical differences between groups were analyzed using one-way analysis of variance (ANOVA) with the least significant difference (LSD) post hoc test using SPSS v21. Statistical differences among the experimental groups are indicated by different alphabetical superscripts, with groups sharing at least one letter (e.g., a and ab) not significantly different, whereas groups with no letters in common (e.g., a, b, and c) differ significantly from each other. A *p*-value < 0.05 was considered statistically significant.

## 3. Results

### 3.1. Fatty Acid Composition of Experimental Diets

As shown in Table 2, the fatty acid composition of the experimental diets varied in the range of SFA content across groups. Total SFA content was lowest in the chow diet (1.5%), with incremental increases in peanut- (5.8%), palmolein- (10.82%), ghee- (15.84%), and coconut oil (21.59%)-enriched HFDs. Total monounsaturated fatty acid (MUFA) content also followed an incremental trend across the groups, except that ghee and coconut showed closely similar MUFA content. Total polyunsaturated fatty acid (PUFA) content was lowest in the ghee- and coconut oil-enriched HFDs. The *n*-6/*n*-3 ratio was lowest in the ghee group compared to other groups, whereas peanut showed the highest ratio. The UFA-to-SFA ratio was lowest in the ghee- and coconut oil-enriched HFDs and highest in the peanut oil-enriched HFD (Table 2).

### 3.2. Food Intake, Body Weight, Organ Weights and Feed Efficiency

Food intake of mice was captured for 16 consecutive weeks after feeding diets enriched with different edible oils and fats. The food intake values are expressed in kcal (Figure 1A,B).

Food intake observed each week differed across the groups, and the number of time points (weeks) with significantly increased food intake also differed across groups compared with the chow diet. For example, food intake was significantly increased (*p* < 0.05) at the 8-, 7-, 5-, and 4-week time points for the peanut, palmolein, coconut, and ghee groups, respectively, compared to the chow diet-fed group. Ghee-fed mice are the only group that showed a significant reduction (*p* < 0.05) in food intake at the 2-week time point compared to chow diet-fed mice (Figure 1B). Again, compared to the ghee diet, calorie intake was significantly (*p* < 0.05) lower at the 5-week time point, indicating that chow-fed mice have the lowest calorie intake among these experimental diets (Figure 1B). At the end of 16 weeks of feeding, the average calorie intake was significantly increased (*p* < 0.05) in the peanut, palmolein, and coconut diets as compared to the chow diet, while it remained insignificant (*p* > 0.05) with ghee-fed mice (Figure 1B). These data indicate preferential food intake in the mice in the order of peanut > palmolein > coconut > ghee as compared to the chow diet, whereas ghee-fed mice showed satiety effects compared to the rest.

Concomitant with food intake, the body weight of mice was also monitored every 10 days and considered as individual data points (Figure 2A,B). The measured body weight of the mice differs across the edible oil-fed diets at each data point. For example, mouse body weight was significantly increased (*p* < 0.05) at the 9-, 7-, 5-, and 4 time points for ghee, coconut, peanut, and palmolein groups, respectively, as compared to the chow diet (Figure 2B).

Although initial body weight remained insignificant across the groups (Table 3), at the end of the 6-month feeding period with different dietary oils/fats, both final body weight and weight gain were significantly increased (*p* < 0.05) in the ghee- and coconut oil-fed groups as compared to the chow-fed group, while the bodyweight changes remained insignificant in the peanut oil-fed group. Although final body weight did not differ significantly, a significant weight gain (*p* < 0.05) was also observed in the palmolein group as compared to the chow-fed group. Feed efficiency ratio (FER) was consistently elevated (*p* < 0.05) in all groups as compared to chow diet-fed mice. However, the FER of ghee-fed mice was the highest (Table 3) among all groups, indicating that these mice convert food into body mass more efficiently than the other groups, which may be due to increased body and organ weights. For example, mice that were fed ghee exhibited a significant and exclusive increase in weight (*p* < 0.05) of metabolically sensitive organs, such as retroperitoneal and epididymal fat (both absolute and relative weight), kidney, and adiposity index, compared to the other dietary groups. Normalized liver weight was significantly elevated (*p* < 0.05) in all dietary groups compared with chow-fed mice, with the highest value in the ghee group (Table 3). In addition to the liver, the kidney weight was marginally increased in coconut oil-fed mice compared with other groups.

### 3.3. Long-Term Intake of SFA-Enriched Diets and Their Effects on Metabolic Parameters Detected in Plasma

Except for peanut, fasting plasma glucose was significantly elevated (*p* < 0.05) in all the groups after 6 months of feeding with different SFA-enriched dietary oils, as compared to chow-fed mice (Figure 3). Ghee-fed mice showed insulin resistance after 6 months, as indicated by a significant insulin spike and elevated HOMA-IR values (*p* < 0.05), whereas peanut- and palmolein-fed mice remained insulin sensitive. Although insulin levels remained insignificant, HOMA-IR was also significantly increased in coconut oil-fed mice compared to the chow group. Plasma cholesterol was significantly elevated across the HFD-fed groups compared with chow-fed mice (*p* < 0.05). However, TG and leptin levels were significantly increased (*p* < 0.05) only in ghee-fed mice among the groups (Figure 3), indicating that most altered metabolic parameters in plasma were observed in ghee-fed mice.

### 3.4. Hepatic Fatty Acid Composition Was Differentially Altered After Feeding Edible Oil-Enriched Diets for Six Months

After feeding HFDs enriched with different edible oils for 6 months, liver fatty acid composition was altered differently across groups compared with the chow diet (Table 4). For example, mice fed a peanut diet exhibited the lowest levels of SFAs in the liver (*p* < 0.05) compared with the other groups. In contrast, hepatic SFA levels were significantly higher (*p* < 0.05) in the ghee and coconut groups compared with the others. Except for the coconut group, total MUFA levels were significantly elevated (*p* < 0.05) in all groups compared with the chow diet. Among all, hepatic *n*-6 PUFA levels were highest (*p* < 0.05), and *n*-3 PUFA levels were lowest (*p* < 0.05) in the peanut group. Moreover, the liver of peanut-fed mice exhibited the highest *n*-6/*n*-3 ratio (15:1), while ghee showed the lowest *n*-6/*n*-3 ratio (Table 4).

### 3.5. Dietary Intake of Ghee and Coconut Oil Significantly Increased Liver TG Levels, Fatty Liver Score, and IL-6 Expression, Whereas Ghee Intake Alone Increased TBARS Level

The morphology of the livers appeared regular in color, with an apparent smooth texture in all the groups (Figure 4A). However, hepatic TG content was significantly elevated in ghee- and coconut oil-fed mice (Figure 4B), although hepatic TC did not differ significantly among the groups (Figure 4C). Histological examination of liver sections stained with H & E revealed a regular hepatic architecture in chow-, peanut-, and palmolein oil-fed mice [Figure 4D(i–iii)]. In contrast, ghee- [Figure 4D(iv)] and coconut oil [Figure 4D(v)]-fed mice exhibited hepatocytes with microvacuoles, focal collections of lymphocytes in the parenchyma, and a moderate number of hepatocytes ballooning, indicating mild microvesicular steatosis. MASLD activity score (MAS) assessment showed a significant increase (~1.3 score), indicating the occurrence of simple steatosis in ghee- and coconut oil-fed mice, but did not develop MASH (Figure 4E). Furthermore, IL6 expression was significantly upregulated (*p* < 0.05) in the mouse livers fed with ghee and coconut oil (Figure 4F, Figure S1), while it remained insignificant in the peanut and palmolein groups, indicating the absence of liver inflammation in the latter groups. Again, the hepatic TBARS level was significantly increased (*p* < 0.05) only in the ghee group compared to others (Figure 4G), indicating oxidative stress in the ghee-fed liver, while no significant changes were observed in glutathione level or catalase and glutathione peroxidase activities among these groups.

### 3.6. Expression of Fatty Acid Oxidative and Lipolytic Genes Was Downregulated in Almost All Edible Oil-Enriched Diets After Feeding for Six Months

mRNA expression of fatty acid oxidation genes, such as *Cpt-1α*, *Pgc-1α* and *Lpl*, was significantly decreased (*p* < 0.05) in the mice fed with diets containing palmolein, ghee, and coconut oils (Table 5). Peanut-fed mice also showed downregulated expression of *Cpt-1α* and *Lpl* mRNA (*p* < 0.05), indicating downregulated beta-oxidation in the liver after six months of feeding dietary oils as compared to the chow diet. Mice fed with a diet containing palmolein showed decreased mRNA expression of *Ppar-α* (*p* < 0.05), whereas in other experimental groups, *Ppar-α* mRNA expression remained unaltered. The expression of *Ppar-γ* remained insignificant among the experimental groups. The expression of antioxidant genes, such as catalase (*Cat*) mRNA levels, was significantly downregulated (*p* < 0.05) in the liver of mice fed with all the dietary oils as compared to the chow diet. Glutathione peroxidase 4 (*Gpx4*) and glutathione reductase (*Gsr*) mRNA levels were significantly downregulated in the liver of mice fed with palmolein and ghee-fed mice compared to those of others.

## 4. Discussion

The present study is the first report to demonstrate that a ghee-enriched diet (25% *w*/*w*) for six months developed mild steatosis and inflammation in the liver by triggering visceral adiposity, insulin resistance, and dyslipidemia in adult mice. Unlike ghee-fed mice, coconut oil-fed mice exhibited hepatic steatosis without increased visceral adiposity, suggesting that insulin resistance may have developed through mechanisms independent of adiposity. In a similar setup, both peanut- and palmolein-fed mice did not develop steatosis, as reflected by lower TG accumulation, lower MAS score, and reduced IL6 expression in the liver, compared with the ghee and coconut oil diets. Although the palmolein diet contains a balanced proportion of SFAs and UFAs, when supplied as a 25% fat source, it showed a mild increase in fasting glucose and bodyweight gain compared to the peanut oil diet. The peanut oil diet exhibited a neutral effect, with no significant changes in fasting glucose, insulin sensitivity, bodyweight gain, adiposity, or TG levels, even though the liver showed a higher *n*-6/*n*-3 fat ratio (15:1) compared to the others. Collectively, these findings indicate that fat type, its composition, dietary level, and metabolic fate can contribute to the development of hepatic steatosis and inflammation in the liver.

The balance of SFAs, MUFAs and PUFAs determines the health effects of vegetable oils/fats. The UFA/SFA ratio, which reflects diet quality, is included in the Healthy Eating Index-2015 [26]. The ghee- and coconut oil-fed groups, which had the lowest UFA/SFA ratio, showed increased liver fat accumulation, higher MAS activity score, and increased liver IL-6 expression, suggesting these oils are more detrimental than palmolein and peanut oil because of their high SFA and low UFA contents. A recent cross-sectional study demonstrated an inverse association between dietary UFA/SFA ratio and liver fibrosis risk [27].

A preferential food intake was observed after six months of feeding with different oils/fats, in the order of peanut > palmolein > coconut > ghee diets, compared with the chow diet. Only ghee-fed mice showed the highest leptin levels and reduced food intake at two time points, indicating a satiety effect. In contrast, bodyweight gain was increased in the order of ghee > coconut > palmolein, while it remained insignificant in peanut oil-fed mice compared with chow-fed mice, indicating a mismatch between food intake and bodyweight gain. Again, feed efficiency ratio (FER) increased in the order of ghee > coconut > palmolein> peanut, indicating that ghee-fed mice absorbed and utilized the diet more efficiently to build organ mass and body mass, as observed by the highest normalized liver weight, adipose fat, kidney, and bodyweight gain compared to the rest. A previous study also showed that mice fed milk fat exhibited higher feed efficiency, bodyweight gain, and adipose tissue content than those fed other animal fat sources [28]. A higher FER may indicate that more of the consumed calories/nutrients are absorbed and converted into tissue or energy storage; improved digestive/absorptive efficiency, as the gut may extract nutrients more effectively or may be burning fewer calories for maintenance; greater fat accumulation; reduced metabolic rate; insulin-related metabolic alterations; or an “energy-saving” phenotype.

Despite the lowest food intake observed in ghee-fed mice, bodyweight gain was the highest among the SFA-fed test groups, indicating that bodyweight gain is influenced more by the source of dietary fat than by the proportion of SFAs in the diet, as shown previously [7]. Our findings are consistent with a previous report demonstrating that, despite reduced or unchanged food intake, an increase in body weight depends on the fat source [7]. Consistent with our observations, a previous study demonstrated that C57BL/6 mice fed with milk fat (15% *w*/*w*) for five weeks exhibited higher feed efficiency, bodyweight gain, and adiposity compared with mice receiving cow tallow, buffalo tallow, mutton tallow, or chicken fat. These results suggest that milk fat is utilized more efficiently by the body than the other fat sources [28].

The positional distribution of fatty acids in the triacylglycerol backbone determines their bioavailability and absorption. Fatty acids in the sn-2 position are absorbed more efficiently than those at the sn-1,3 position [29]. Ghee, a typically anhydrous milk fat, contains triacylglycerol with palmitic acid partly at the sn-2 position, and during digestion, pancreatic lipase selectively hydrolyzes the sn-1 and sn-3 fatty acids, producing sn-2 monoacylglycerol (MAG), which is efficiently absorbed and re-esterified [30]. However, the impact of sn-2 palmitate-derived absorption on lipid transport and hepatic TG accumulation in this study remains unclear. Although we did not measure it, data suggest that bovine milk fat like ghee contains a relatively high proportion of palmitic acid at the sn-2 position [31,32] because of the way milk fat is biosynthesized in the mammary gland of ruminants. The excess sn-2 MAG in the ghee diet might have been directly incorporated into chylomicron TGs, thereby increasing lipid accumulation in the liver. Thus, compared to eucaloric peanut- and palmolein-fed mice, ghee-fed mice might have received a different quality of fats that contain a greater proportion of SFAs acylated at the sn-2 position than the vegetable oils used in this study, which predominantly contain UFAs in the same position.

Despite no increase in mean caloric intake in ghee-fed mice (Figure 1), they still showed accumulation of serum and hepatic TGs, accompanied by liver steatosis. Similar to a previous study in Fischer inbred rats fed with a 10% ghee diet for four weeks [18], the current study with a 25% ghee diet also reported an increase in serum TGs. A previous study showed that a higher SFA diet is more obesogenic than diets with lower SFA content [33]. However, in this study, a higher adiposity was observed in lower SFA-enriched ghee-fed mice than in coconut (Table 2), which could be due to differences in fat sources (milk fat vs. vegetable oil), species (SD rats vs. C57 mice), duration (50 days vs. 180 days), and composition of the diet between the study designs.

The greater weight gain and excess hepatic lipid accumulation observed with SFA-rich diets in this study may also be associated with increased availability of endogenous fatty acids and their metabolites to the gut microbiota [34]. Dietary SFAs promote greater weight gain and hepatic lipid accumulation than UFAs, potentially because their lower absorption efficiency in the proximal intestine allows more fat to reach the distal intestine, where it alters the gut microbiota in ways that promote inflammation, metabolic dysfunction, and increased lipid deposition in adipose tissue and the liver [6]. In particular, diets richer in SFAs increased the abundance of Firmicutes and reduced the abundance of Bacteroidetes; for example, canola oil (lower SFA content) produced the smallest microbiota shifts, whereas lard (higher SFA content) induced the greatest changes. These findings suggest that the elevated hepatic TG levels and lipid accumulation observed in SFA-enriched ghee- and coconut oil-fed mice may be mediated, at least in part, by metabolites produced by the altered gut microbiota.

Unlike the ghee diet, the coconut oil diet promoted insulin resistance and lipid accumulation in the liver independent of visceral adiposity. Like ghee-fed mice, a HOMA-IR together with elevated glucose suggests insulin resistance, even if fasting insulin itself was not different. Since HOMA-IR incorporates both fasting glucose and insulin, the observed increase in HOMA-IR appears to be driven primarily by higher fasting glucose levels and reflects altered insulin sensitivity that was not evident from fasting insulin measurements alone. The development of insulin resistance in coconut oil-fed mice may be attributable to excess hepatic lipid accumulation. Because the sn-2 position of coconut oil triacylglycerols is enriched with lauric (C 12:0) and myristic acids (C 14:0), as reported in [32], these SFAs accumulated to a greater extent in the liver of coconut oil-fed mice (Table 4). Unlike shorter medium-chain fatty acids (C8:0 and C10:0), a considerable proportion of lauric acid is re-esterified into TGs within enterocytes and incorporated into chylomicrons, allowing transport through the lymphatic circulation and delivery to the liver. Excess hepatic uptake of lauric acid might therefore have promoted TG accumulation in the liver, leading to hepatic insulin resistance, even in the absence of visceral adiposity in this study. SFAs present in coconut oil, particularly lauric acid and other medium-chain SFAs, have been reported to induce inflammatory signaling, endoplasmic reticulum stress, and defects in insulin signaling pathways in metabolic tissues. Consequently, insulin resistance may develop before or independently of detectable visceral fat accumulation [19]. Thus, the observed increase in HOMA-IR despite unchanged visceral adiposity may reflect liver-specific metabolic dysfunction rather than obesity-driven insulin resistance.

In this study, palmolein-fed mice exhibited a modest increase in weight gain without insulin resistance, in contrast to several reports showing palm oil-induced insulin resistance and glucose intolerance at different energy levels [10,35,36]. Consistent with a study at a comparable fat level (20%) with oleic acid-rich palmolein in Wistar rats, our study also observed a mild increase in fasting glucose after 24 weeks. However, fasting insulin remained unchanged, in contrast to the earlier finding of hyperinsulinemia [37]. These findings suggest that while a long-term palmolein-enriched diet (25%) can promote weight gain and mild disturbances in glucose homeostasis, its effects on insulin regulation may vary depending on the specific fatty acid composition of the oil used (44.3% oleic acid and 39.5% palmitic acid vs. 44.9% oleic acid and 35.87% palmitic acid), duration of exposure (21 vs. 24 weeks), and species (rat vs. mouse). Unlike the previous study, where palm oil in HFD (41% fat) fed for 8 weeks in C57Bl/6J mice disrupted the blood lipid profile, accumulated liver fats and caused hepatocyte ballooning [38], a six-month palmolein oil-enriched diet (25% fat) in this study did not induce hepatic steatosis and liver inflammation in similar mice, possibly due to the difference in fat intake. Unlike this work, a palm oil-based diet with comparable fat (23.8%) showed hepatic steatosis in C57BL/6 mice after 8 weeks [10], which could be due to differences in the fatty acid composition of diets (palm oil vs. palmolein) and the adapted metabolic response over time (8 vs. 24 weeks).

A two-month crossover trial comparing palmolein and olive oil (48 g per day) showed no differences in body weight, blood lipids, glucose, or insulin in healthy adults [39]. Again, in a 16-week randomized controlled trial, diets enriched with palmolein, cocoa butter, or soybean oil had no differential effect on liver fat concentration when total dietary fat was maintained at 33% of energy [15], indicating inconsistency in the effect of palmolein intake on hepatic lipid accumulation. Irrespective of the composition of dietary oils, the metabolic impact of oils on liver fats might depend on the absolute energy of diets derived from fat sources. For example, compared to the control diet (11% energy from fat), both HFDs rich in palm and olive oil (56% energy from fat) intake showed hepatic lipid accumulation in male Wistar rats after 12 weeks [36], indicating that olive oil in excess could have an equal adverse impact on liver fat metabolism as palm oil.

In palmolein, most SFAs are in the sn-1,3 position, whereas UFAs (80%) are in the sn-2 position [40]. Although total SFA content was similar, mice fed a palmolein oil diet showed decreased fat deposition and increased fecal excretion of SFAs compared to interesterified palmolein in which the sn-2 position was esterified with SFAs [41]. A controlled metabolic study showed that in healthy adults, consuming SFA-enriched palmolein and coco butter with most SFAs in the sn-1,3 position did not alter liver fat concentration or adipose tissue weight [15]. In the present study, compared with SFA-enriched ghee and coconut oil, palmolein did not increase liver fat content, possibly because of the positional distribution of SFAs in the sn-1 and sn-3 positions of the triacylglycerol backbone. The lack of adverse effects in peanut-fed mice compared with coconut- and ghee-fed mice may also reflect the higher MUFA content of peanut oil, with MUFAs preferentially incorporated into hepatic TGs (Table 4). The liver might actively channel fatty acids into membrane phospholipids and TGs to preserve membrane fluidity, consistent with evidence that MUFAs are preferentially esterified into TGs and phospholipids and are less lipotoxic than SFAs [42]. Moreover, lipid-synthesizing enzymes, such as monoacyl- and diacylglycerol acyltransferases, prefer unsaturated acyl chains over saturated ones during TG synthesis. The increase in MUFA levels in peanut- and palmolein-fed liver (Table 4) might have prevented the accumulation of hepatic TGs by stabilizing TG storage. Since TGs containing MUFAs remain more fluid and are easier to package into lipid droplets [43], this may drive such physiological alterations. All HFDs increased TC in blood plasma, possibly due to upregulation of HMG-CoA reductase activity, as reported earlier [44].

Following six months of dietary intervention, the reductions in hepatic *Cpt1α*, *Pgc1α*, and *Cat* mRNA expression across all HFD groups suggest shared alterations in fatty-acid oxidation and antioxidant defense genes. However, these changes failed to explain why hepatic TG accumulation and steatosis were predominant in the ghee- and coconut oil-fed groups. Long-term HFD exposure can produce time-dependent hepatic adaptations, with late-stage changes involving lipid uptake, de novo lipogenesis, and lipid storage, alongside alterations in oxidative pathways [45,46]. Although increased expression of oxidative genes can represent an adaptive response to excess dietary fat, the reduced expression observed in this study may indicate an altered oxidative response after chronic feeding; however, transcript levels alone cannot establish fatty-acid oxidation rates. Reduced hepatic *Lpl* mRNA expression observed in the present study may be linked to altered lipid handling in hepatic tissue, as lipid accumulation is determined by an imbalance between lipid acquisition (fatty acid uptake and de novo lipogenesis) and disposal (fatty acid oxidation and VLDL secretion), such that increased fatty acid delivery and/or reduced lipid disposal may contribute to hepatic steatosis independently of changes in *Lpl* expression [47,48].

Liver TG levels were elevated in the ghee and coconut groups, as supported by histological evidence of microvesicular steatosis, inflammatory mediators, and increased MAS scores. Liver fatty acid composition further demonstrated increased SFA content in these groups, supporting the role of SFA-rich diets in hepatic fat accumulation. Consistent with our findings, previous studies have shown increased liver TGs and steatosis in ghee- and coconut oil-fed rodents [20,28]. In contrast, the palmolein diet did not increase hepatic lipid content in our study, aligning with reports indicating minimal hepatic fat accumulation at similar intake levels [36]. However, palmolein intake in an HFD setup (41 to 45% kcal from fat) has been associated with hepatic steatosis and hepatocyte ballooning [16,38]. The markedly elevated circulating leptin levels in ghee-fed mice may primarily reflect increased adiposity rather than a sustained satiety effect, as reduced food intake was observed only at two time points and was not maintained throughout the intervention. Given the association between obesity-related hyperleptinemia and impaired leptin signaling, these findings may also suggest leptin resistance, although this requires direct functional assessment [49,50]. In addition to increased adiposity, insulin resistance, and higher MASLD activity score (MAS) in the ghee and coconut groups, elevated TBARS levels in the liver indicate enhanced oxidative stress in these groups. Additionally, a relative increase in IL-6 expression further confirms that the liver was inflamed in addition to accumulating fat.

The present dietary trial confirms long-term changes in the metabolically sensitive organ, such as the liver, after 6 months of feeding with different SFA-enriched diets, as reflected by the metabolic trace of fatty acids (Table 2) and their proportionate incorporation into the liver (Table 4). For example, SFA-enriched coconut- and ghee-fed mice result in a higher incorporation of total SFAs in the liver as compared to other diets. Similarly, a relative proportion of MUFAs in the peanut and palmolein diets was also incorporated proportionately in the livers compared to the chow diet. Again, total *n*-6 PUFA levels were elevated in the peanut group and decreased in the palmolein-fed mice. Such dietary incorporation of fatty acids into the liver could be due to differences in dietary lipid content, preferential incorporation of UFAs, or increased desaturation of SFAs in the liver. Overall, the present dietary trial established a defined model for assessing the metabolic changes induced by dietary modification.

A key limitation of this study is that only male mice were included, and sex-specific differences in metabolic responses to HFDs may influence the effects of different dietary fat sources; therefore, the findings cannot be directly extrapolated to females. Due to limited plasma and liver tissue, the study could not evaluate circulating IL-6 levels or additional inflammatory markers. Although both phenotypic and biochemical indices clearly established metabolic disruption of liver homeostasis, the study is limited in explaining the mechanisms underlying coconut- and ghee-induced hepatic disruption at the molecular level. The study did not include other dietary oils, such as sunflower, rice bran, and soybean, which have SFA contents similar to those of the peanut and palmolein oils used in this study.

## 5. Conclusions

Despite the low food intake, the ghee diet (25% *w*/*w*) increased bodyweight gain, visceral adiposity, and insulin resistance, making it more susceptible to developing fatty liver than the other diets used in this study. Although the relative bodyweight gain was increased in all high-SFA-fed mice, adiposity was only increased in ghee-fed mice, indicating that increased visceral fat may predispose to the development of fatty liver. Among the sets of SFAs containing oils chosen in this dietary intervention trial, ghee- and coconut oil-enriched diets selectively induced mild microvesicular steatosis and inflammation in mouse liver compared with the peanut and palmolein diets. Therefore, the results may support the conclusion that palmolein and peanut oils were less detrimental than ghee or coconut oil under these experimental conditions. However, further work is required to confirm whether recommended intake of fats (20–30% of total calories) for a similar duration yields similar effects in the liver. Collectively, these findings indicate that the source of dietary fat, degree of saturation, and fatty acid composition of the diets may play a crucial role in the development of metabolic risk factors such as obesity, impaired glucose metabolism, dyslipidemia, and hepatic steatosis.

## Figures and Tables

**Figure 1:**
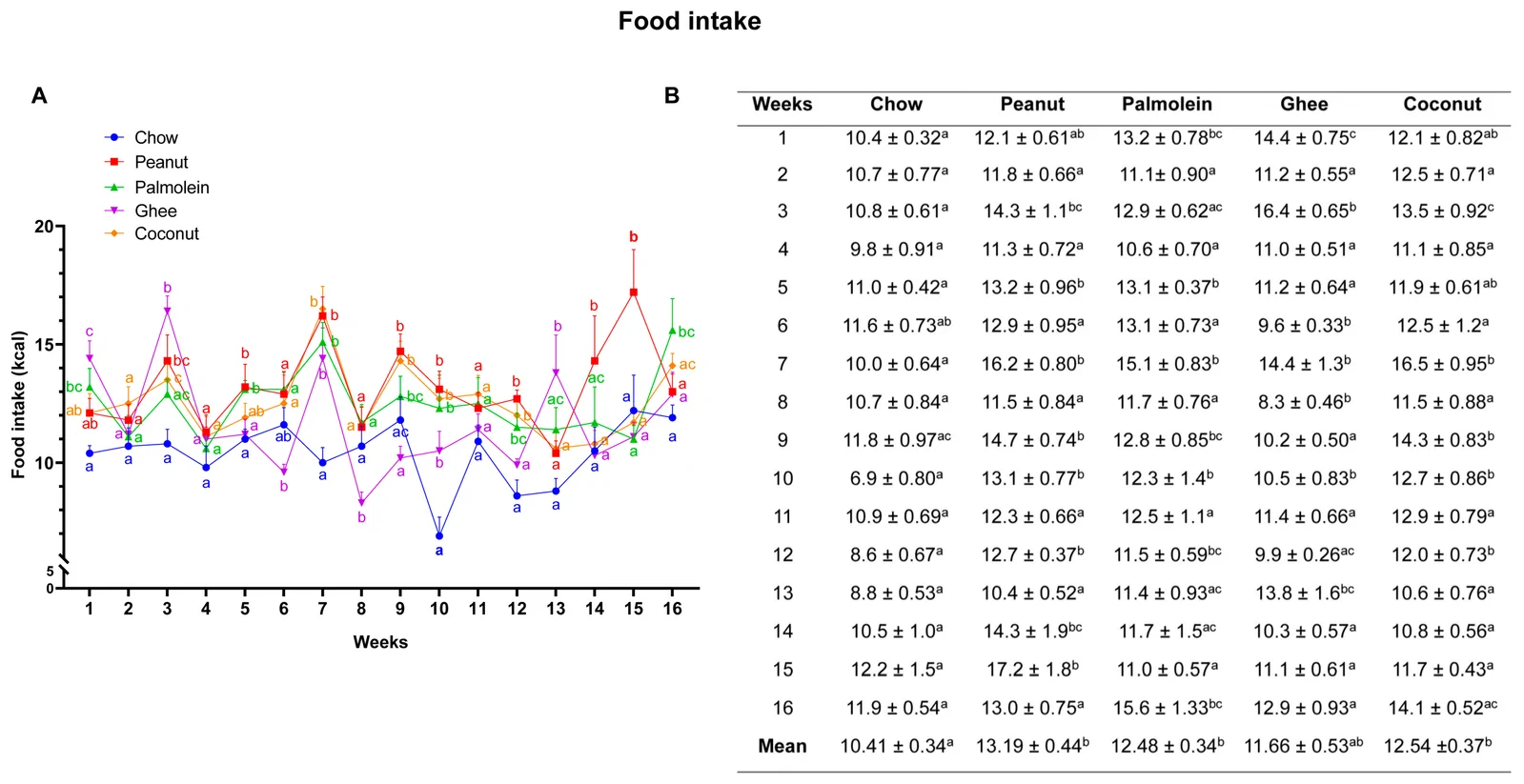
Food intake of mice measured weekly during six months of feeding with HFDs enriched with different proportions of SFAs from dietary oils. Food intake was estimated based on leftovers and expressed in kcal. (**A**) The intake pattern of mice is plotted across 16 weeks. (**B**) The corresponding weekly kcal values are tabulated for the experimental groups. Values are represented as mean ± SEM (*n* = 8 mice per group). Data were analyzed by one-way ANOVA, and statistical significance was considered at *p* < 0.05. Different superscript letters indicate significant differences among the groups.

**Figure 2:**
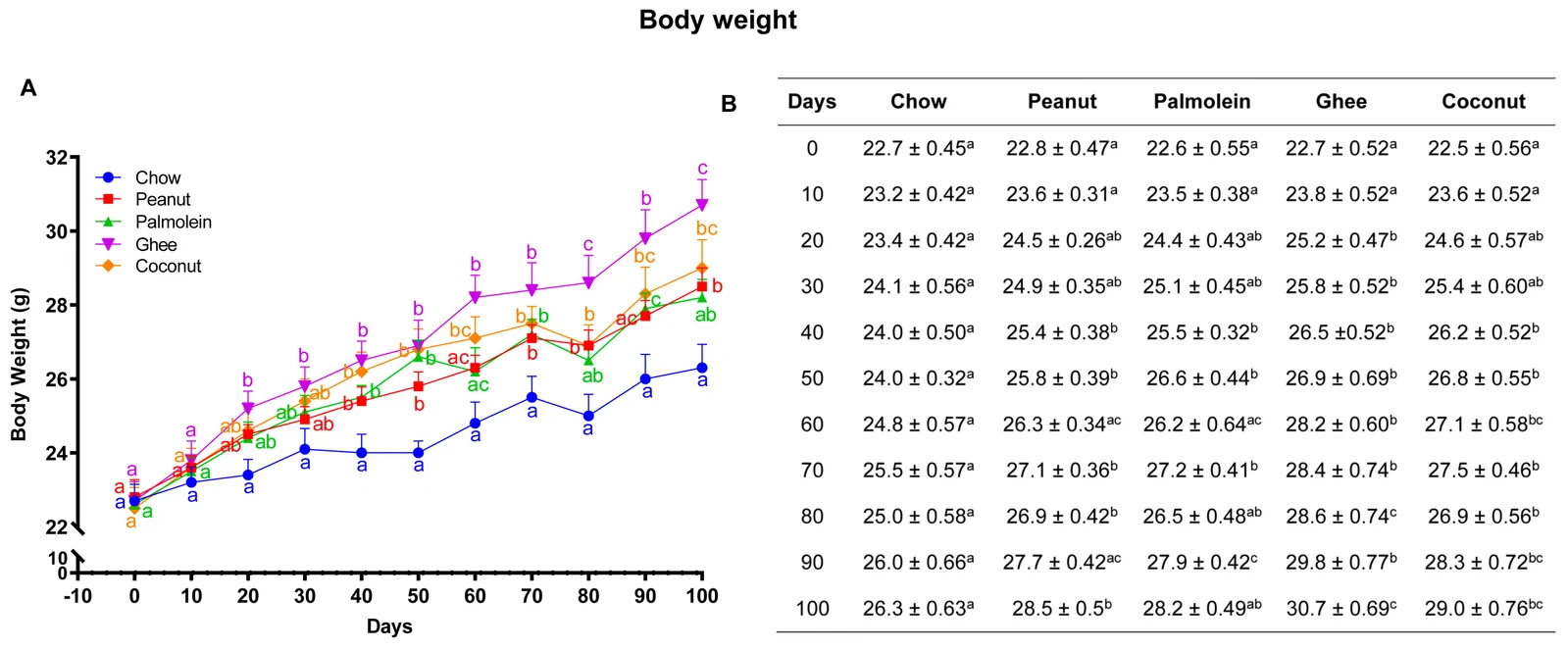
(**A**,**B**) Body weight of mice was measured every ten days during feeding with HFD enriched with different proportions of SFAs from dietary oils. Data are expressed in grams (g). Values are represented as mean ± SEM (*n* = 8 mice per group). Data were analyzed by one-way ANOVA, and statistical significance was considered at *p* < 0.05. Different superscript letters indicate significant differences among the groups.

**Figure 3:**
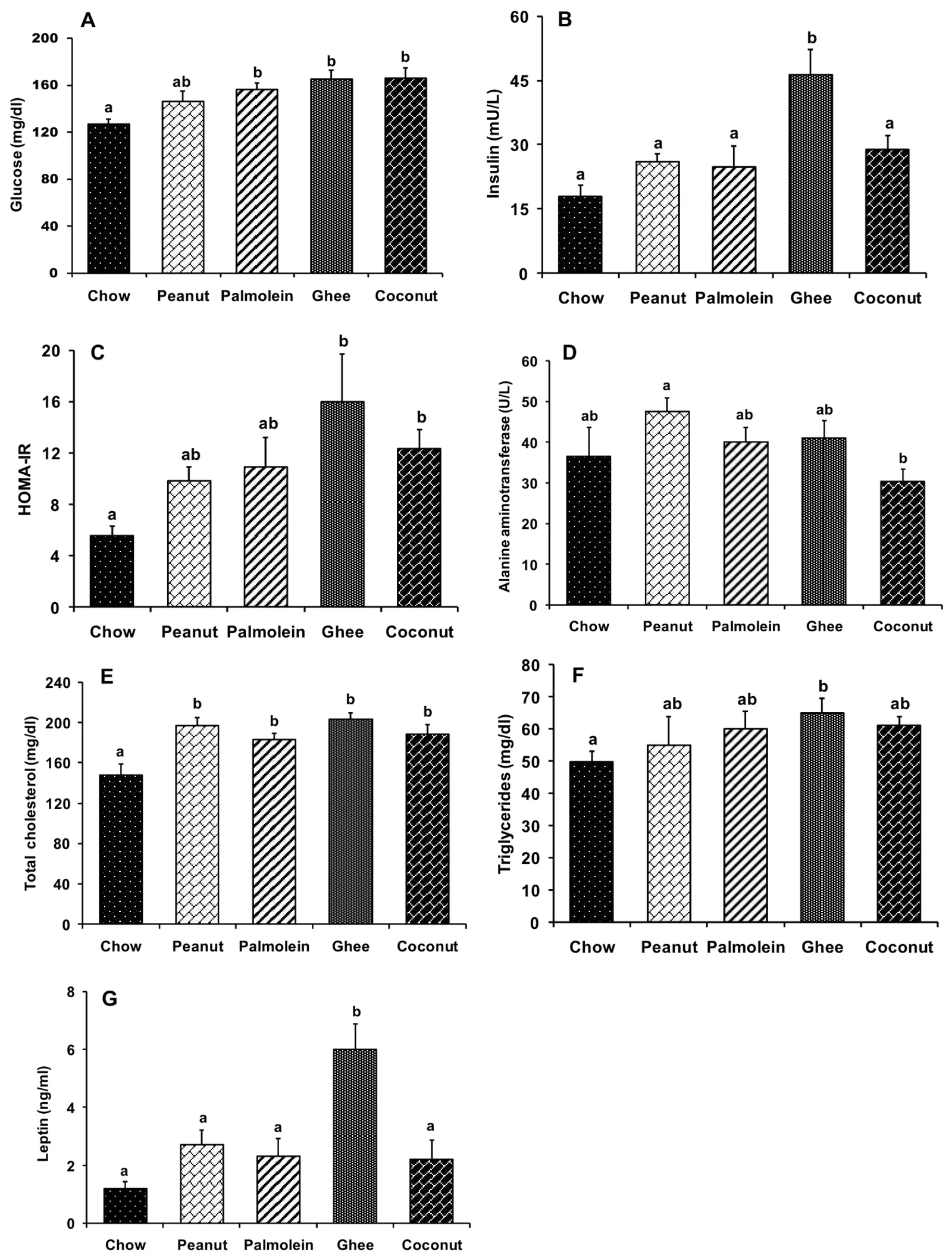
Long-term (six-month) intake of SFA-enriched dietary oils on metabolic parameters measured in plasma. (**A**) Glucose, (**B**) insulin, (**C**) HOMA-IR, (**D**) alanine aminotransferase, (**E**) total cholesterol (TC), (**F**) triglycerides (TGs), and (**G**) leptin. Values are represented as mean ± SEM (*n* = 8 mice per group). Different superscript letters indicate significant differences among the groups at *p* < 0.05.

**Figure 4:**
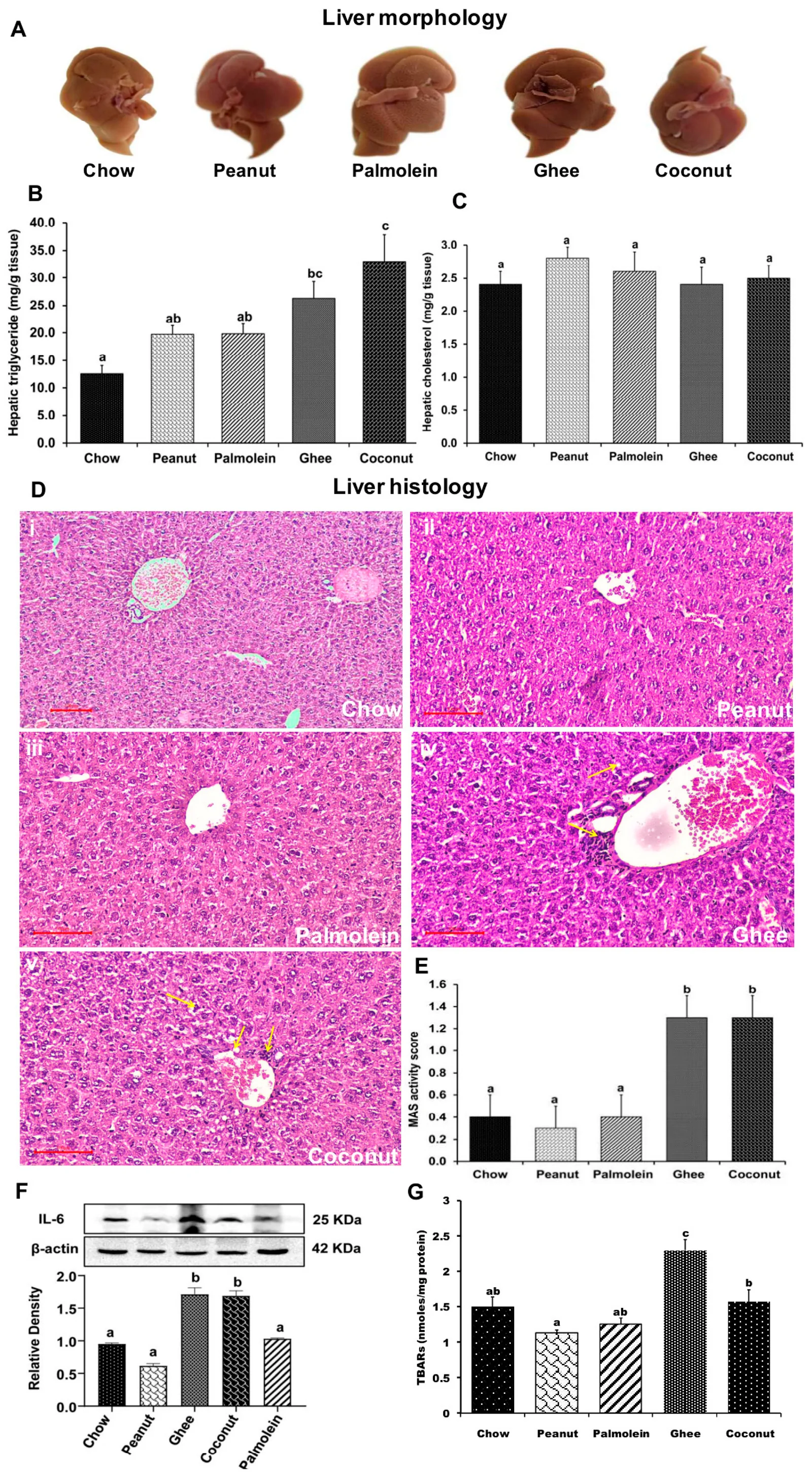
Effects of dietary intake of SFA-enriched oils on liver phenotype, lipid content, inflammation, oxidative stress and fatty liver score after six months of feeding in mice. (**A**) Representative liver morphology of mice fed with different dietary fats, (**B**) hepatic TG content, and (**C**) hepatic TC content. (**D**) Microphotograph showing liver histology with H & E staining (20X magnification, scale bar = 100 µm) of different dietary groups (**i**–**v**), as indicated in the images. Yellow arrows indicate micro vacuolation and focal collection of lymphocytes. (**E**) MASLD activity score (MAS). Values are represented as mean ± SEM (*n* = 8 mice per group). (**F**) Immunoblots of IL-6 show the expression of the protein and respective quantitative changes in protein levels expressed as the relative band density after being normalized with β-actin protein expression. The data are presented as mean ± SEM (*n* = 6 mice/group) derived from three independent experiments. (**G**) TBARS (*n* = 8 mice per group). Data were analyzed by one-way ANOVA, and statistical significance was considered at *p* < 0.05. Different superscript letters indicate significant differences among the groups. Values with dissimilar letters show significant differences at *p* < 0.05 vs. control.

**Table 1:** Composition of chow diet (100 g).

Ingredients	Quantity (g)
Roasted Bengal gram	59.0
Wheat	22.5
Casein	4.0
Skim milk powder	5.0
Groundnut oil	5.0
Vitamin mix	0.5
Salt mix	4.0

**Table 2:** Fatty acid composition of the experimental diets (g/100 g).

Fatty Acids	Chow	Peanut	Palmolein	Ghee	Coconut
C12:0	0.01	0.01	0.07	0.68	9.39
C14:0	0.02	0.04	0.29	2.78	7.07
C16:0	1.07	4.06	9.21	9.14	3.87
C18:0	0.25	0.96	1.25	3.24	1.26
C22:0	0.15	0.73	-	-	-
Total SFAs	1.50	5.80	10.82	15.84	21.59
C14:1	-	-	-	0.41	-
C16:1	0.01	0.03	0.05	0.61	0.01
C18:1	2.74	11.61	11.62	8.07	3.30
Total MUFAs	4.25	17.44	22.49	24.93	24.90
C18:2*n*-6	2.70	9.06	4.01	1.52	1.65
C18:3*n*-3	0.06	0.07	0.09	0.15	0.04
Total PUFAs	2.76	9.13	4.10	1.67	1.69
UFAs/SFAs	4.67	4.58	2.46	1.68	1.23
*n*-6:*n*-3	45.00	129.43	44.55	10.13	41.25

SFAs, saturated fatty acids; MUFAs, monounsaturated fatty acids; PUFAs, polyunsaturated fatty acids; UFAs, unsaturated fatty acids.

**Table 3:** Murinometric characterization of metabolically sensitive organs after six months of intake of SFA-enriched dietary oils ^#^.

Parameters	Chow	Peanut	Palmolein	Ghee	Coconut
Initial body weight (g)	22.7 ± 0.45 ^a^	22.8 ± 0.47 ^a^	22.6 ± 0.55 ^a^	22.7 ± 0.52 ^a^	22.5 ± 0.56 ^a^
Final body weight (g)	28.4 ± 0.97 ^a^	30.6 ± 0.84 ^ac^	31.1 ± 1.0 ^ab^	33.7 ± 1.2 ^b^	31.6 ± 1.0 ^bc^
Weight gain (g)	5.7 ± 0.68 ^a^	7.8 ± 0.91 ^ab^	8.5 ± 0.87 ^b^	11.0 ± 1.1 ^c^	9.1 ± 0.84 ^bc^
Cumulative energy intake (kcal/group)	79.04	105.34	103.00	96.31	103.11
Feed efficiency ratio (%)	1.1 ± 0.12 ^a^	1.6 ± 0.18 ^b^	1.7 ± 0.17 ^b^	2.4 ± 0.21 ^c^	1.9 ± 0.16 ^b^
Liver (% bw)	4.2 ± 0.06 ^a^	4.9 ± 0.14 ^bc^	4.6 ± 0.08 ^c^	5.1 ± 0.24 ^b^	4.9 ± 0.11 ^bc^
Kidney (% bw)	1.2 ± 0.02 ^ac^	1.1 ± 0.02 ^a^	1.2 ± 0.02 ^cd^	1.4 ± 0.04 ^b^	1.3 ± 0.04 ^bd^
Absolute RP (g)	0.09 ± 0.014 ^a^	0.19 ± 0.051 ^ab^	0.19 ± 0.055 ^ab^	0.32 ± 0.067 ^b^	0.16 ± 0.043 ^a^
Relative RP (% bw)	0.31 ± 0.05 ^a^	0.60 ± 0.15 ^ab^	0.58 ± 0.15 ^ab^	0.91 ± 0.18 ^b^	0.49 ± 0.11 ^a^
Absolute EP (g)	0.54 ± 0.055 ^a^	0.67 ± 0.083 ^a^	0.82 ± 0.12 ^ab^	1.13 ± 0.15 ^b^	0.79 ± 0.13 ^a^
Relative EP (% bw)	1.9 ± 0.15 ^a^	2.1 ± 0.23 ^a^	2.6 ± 0.29 ^ab^	3.3 ± 0.37 ^b^	2.4 ± 0.33 ^a^
Adiposity index (%)	8.0 ± 0.53 ^a^	8.8 ± 0.97 ^a^	10 ± 1.0 ^ab^	12.3 ± 1.3 ^b^	9.1 ± 1.1 ^a^

^#^ Values represented in mean ± SEM (*n* = 8 mice per group). Data were analyzed by one-way ANOVA, and statistical significance was considered at *p* < 0.05. Different superscript letters indicate significant differences among the groups. bw: body weight. g: gram. RP: retroperitoneal fat; EP: epididymal fat. Feed efficiency ratio is expressed as a percentage, calculated as the ratio of total bodyweight gain (g/day) to total food intake (g/day).

**Table 4:** Effects of SFA-enriched dietary oil intake on liver fatty acid composition (*n* mole %) ^#^.

Fatty Acids	Chow	Peanut	Palmolein	Ghee	Coconut
C12:0	0.27 ± 0.02 ^a^	0.12 ± 0.02 ^a^	0.22 ± 0.29 ^a^	0.19 ± 0.02 ^a^	2.5 ± 0.58 ^b^
C14:0	0.71 ± 0.07 ^ab^	0.39 ± 0.03 ^a^	0.49 ± 0.04 ^a^	1.35 ± 0.08 ^b^	4.6 ± 0.50 ^c^
C16:0	25.0 ± 0.47 ^a^	22.3 ± 0.69 ^b^	27.8 ± 0.56 ^c^	30.7 ± 0.34 ^d^	27.0 ± 0.59 ^c^
C18:0	11.9 ± 0.83 ^a^	10.3 ± 0.24 ^ab^	11.3 ± 0.33 ^ab^	10.1 ± 0.52 ^b^	11.3 ± 0.72 ^ab^
C20:0	0.26 ± 0.011 ^a^	0.32 ± 0.007 ^ab^	0.32 ± 0.036 ^ab^	0.44 ± 0.025 ^c^	0.35 ± 0.022 ^b^
C24:0	0.19 ± 0.019 ^ac^	0.18 ± 0.007 ^ac^	0.21 ± 0.05 ^bc^	0.11 ± 0.01 ^a^	0.14 ± 0.03 ^ab^
Total SFAs	38.4 ± 1.1 ^a^	33.6 ± 0.9 ^b^	40.3 ± 0.67 ^a^	43.0 ± 0.32 ^c^	45.5 ± 0.63 ^d^
C16:1	1.4 ± 0.19 ^a^	0.37 ± 0.03 ^b^	0.72 ± 0.12 ^b^	2.0 ± 0.19 ^c^	2.2 ± 0.32 ^c^
C18:1	17.1 ± 1.5 ^a^	22.0 ± 0.60 ^b^	23.0 ± 1.27 ^b^	22.9 ± 0.97 ^b^	16.6 ± 0.95 ^a^
Total MUFAs	18.5 ± 1.7 ^a^	22.4 ± 0.60 ^bc^	23.7 ± 1.3 ^c^	24.9 ± 1.1 ^c^	18.8 ± 1.3 ^ab^
C18:2*n*-6	22.1 ± 1.0 ^a^	26.8 ± 0.66 ^b^	17.4 ± 0.35 ^ce^	16.5 ± 0.17 ^c^	16.9 ± 0.34 ^cd^
C20:4*n*-6	13.8 ± 1.1 ^a^	12.7 ± 0.4 ^a^	12.6 ± 0.73 ^a^	9.7 ± 0.5 ^b^	11.9 ± 1.2 ^ab^
C22:4*n*-6	0.35 ± 0.05 ^a^	0.38 ± 0.016 ^a^	0.28 ± 0.06 ^c^	0.19 ± 0.006 ^b^	0.32 ± 0.08 ^ab^
C22:5*n*-6	1.39 ± 0.28 ^ac^	1.2 ± 0.019 ^ac^	0.97 ± 0.10 ^c^	0.37 ± 0.03 ^b^	1.6 ± 0.23 ^a^
Total *n*-6 PUFAs	37.7 ± 0.51 ^a^	41.1 ± 0.43 ^b^	31.3 ± 0.76 ^de^	26.8 ± 0.59 ^c^	30.7 ± 1.2 ^d^
C18:3*n*-3	0.18 ± 0.02 ^a^	0.10 ± 0.01 ^b^	0.10 ± 0.02 ^a^	0.23 ± 0.014 ^a^	0.13 ± 0.014 ^b^
C22:5*n*-3	0.2 ± 0.03	ND	0.14 ± 0.03	0.46 ± 0.02	0.29 ± 0.03
C22:6*n*-3	5.1 ± 0.36 ^a^	2.6 ± 0.04 ^b^	4.1 ± 0.34 ^c^	4.5 ± 0.27 ^ac^	4.4 ± 0.35 ^ac^
Total *n*-3 PUFAs	5.4 ± 0.34 ^a^	2.7 ± 0.03 ^b^	4.3 ± 0.36 ^c^	5.2 ± 0.27 ^ac^	4.7 ± 0.39 ^ac^
*n*-6/*n*-3	7.1 ± 0.49 ^a^	15.1 ± 0.26 ^b^	7.4 ± 0.51 ^ae^	5.2 ± 0.16 ^c^	6.7 ± 0.59 ^ad^

^#^ Values represented as mean ± SEM (*n* = 8/group). Data were analyzed by one-way ANOVA, and statistical significance was considered at *p* < 0.05. Different superscript letters indicate significant differences among the groups. ND: not detected.

**Table 5:** mRNA levels of fatty acid oxidation, lipolytic and antioxidant genes expressed in hepatic tissues of mice fed with dietary oils containing different fat sources.

Gene Pathway	Gene Symbol	Relative mRNA Fold Expression ^#^ (Target Gene/*Actb*)				
		Chow	Peanut	Palmolein	Ghee	Coconut
Fatty acid β-oxidation	*Ppar-α*	1.02 ± 0.11 ^a^	0.84 ± 0.11 ^a^	0.66 ± 0.06 ^b^	0.79 ± 0.02 ^a^	0.84 ± 0.05 ^a^
	*Ppar-γ*	1.01 ± 0.10 ^a^	0.65 ± 0.13 ^a^	0.87 ± 0.19 ^a^	0.75 ± 0.16 ^a^	0.96 ± 0.19 ^a^
	*Cpt-1α*	1.03 ± 0.13 ^a^	0.61 ± 0.09 ^b^	0.11 ± 0.06 ^b^	0.54 ± 0.07 ^b^	0.59 ± 0.11 ^b^
	*Pgc-1 α*	1.01 ± 0.08 ^a^	0.70 ± 0.15 ^a^	0.25 ± 0.13 ^b^	0.44 ± 0.04 ^b^	0.54 ± 0.08 ^b^
	*Lpl*	1.01 ± 0.07 ^a^	0.67 ± 0.12 ^b^	0.66 ± 0.06 ^b^	0.64 ± 0.05 ^b^	0.54 ± 0.07 ^b^
Antioxidant	*Cat*	1.01 ± 0.10 ^a^	0.53 ± 0.13 ^b^	0.32 ± 0.10 ^b^	0.30 ± 0.07 ^b^	0.28 ± 0.06 ^b^
	*Gpx4*	1.01 ± 0.09 ^a^	0.66 ± 0.12 ^a^	0.26 ± 0.11 ^b^	0.47 ± 0.10 ^b^	0.62 ± 0.12 ^a^
	*Gsr*	1.03 ± 0.11 ^a^	0.76 ± 0.12 ^a^	0.63 ± 0.06 ^b^	0.61 ± 0.04 ^b^	0.75 ± 0.04 ^a^

^#^ Relative mRNA expression levels of each gene in mice liver tissue are expressed as a relative fold expression after normalizing their expression with endogenous control, β-Actin (*Actb*). Data are analyzed with one-way ANOVA using Dunnett’s multiple comparison test. Values are represented as means ± SEM (*n* = 6 mice/group, with each mouse analyzed in duplicate). Values with unlike letters were significantly different *p* < 0.05 vs. control.

## Data Availability

The data presented in this study are available on request from the first and corresponding authors.

## Supplementary Materials

The following supporting information can be downloaded at: https://www.mdpi.com/article/10.3390/nu18213642/s1, Figure S1. Expression of IL-6 in the mice liver after 6 months of feeding with diets containing different oils.

## Abbreviations

The following abbreviations are used in this manuscript:

AIN 93M	American Institute of Nutrition-1993 maintenance
cDNA	Complementary deoxyribonucleic acid
CPT-1α	Carnitine Palmitoyl transferase 1A
Ct	Threshold cycle
ECL	Enhanced chemiluminescence
EDTA	Ethylenediaminetetraacetic acid
ELISA	Enzyme-Linked Immunosorbent Assay
gDNA	Genomic deoxyribonucleic acid
HDL	High-density lipoprotein
HFD	High-fat diet
HOMA-IR	Homeostatic model assessment of insulin resistance
HRP	Horseradish peroxidase
IgG	Immunoglobulin G
IL6	Interleukin 6
kcal	kilocalorie
KO	Knock-out
LDL	Low-density lipoprotein
LPL	Lipoprotein lipase
MASH	Metabolic dysfunction-associated steatohepatitis
MASLD	Metabolic dysfunction-associated steatotic liver disease
mRNA	Messenger ribonucleic acid
MUFA	Monounsaturated fatty acid
ND	Not detected
PBS	Phosphate-buffered saline
PGC-1α	Peroxisome proliferator-activated receptor gamma coactivator 1-alpha
PPARα	Peroxisome proliferator-activated receptor (PPAR)-alpha
PUFAs	Polyunsaturated fatty acids
RIPA	Radioimmunoprecipitation assay
RT-qPCR	Quantitative reverse transcription polymerase chain reaction
SDS-PAGE	Sodium dodecyl sulfate–polyacrylamide gel electrophoresis
SEM	Standard error of the mean
SFA	Saturated fatty acid
sn	stereo-specific number
TBARS	Thiobarbituric acid reactive substances
TC	Total cholesterol
TG	Triglyceride
UFAs	Unsaturated fatty acids

## Appendix A

**Table A1:** Fatty acid composition of dietary fat sources (%).

Fatty Acids	Peanut	Palmolein	Ghee	Coconut
C12:0	0.03	0.26	2.72	37.55
C14:0	0.12	1.10	11.06	28.24
C16:0	15.27	35.87	35.56	14.49
C18:0	3.66	4.80	12.78	4.83
C22:0	2.93	ND	ND	ND
Total SFA	22.02	42.03	62.11	85.11
C14:1	ND	ND	1.63	ND
C16:1	0.09	0.18	2.40	ND
C18:1	44.86	44.91	30.69	11.62
Total MUFA	44.94	45.09	34.73	11.62
C18:2	32.93	12.71	2.75	3.28
C18:3	0.11	0.18	0.41	ND
Total PUFA	33.04	12.88	3.16	3.28

**Table A2:** Gene pathways and predesigned SYBR green I primers used for the mRNA expression analyses of mice liver samples.

Primer ID	Gene Symbol	Gene ID	Gene Name	Nucleotide Sequences (5′-3′)	Ref. seqID
Fatty acid oxidative and lipolytic genes					
M_Ppara_1	*Ppar-α*	19013	Peroxisome proliferator activated receptor alpha	F 5′-GATGTCACACAATGCAATTC-3′ F 5′-CAGTTTCCGAATCTTTCAGG-3′	NM_011144
M_Pparg_1	*Ppar-γ*	19016	Peroxisome proliferator activated receptor gamma	F 5′-AAAGACAACGGACAAATCAC-3′ R 5′-GGGATATTTTTGGCATACTCTG-3′	NM_001127330
M_Cpt1a_1	*Cpt-1 α*	12894	Carnitine Palmitoyl transferase 1a	F 5′-GGGAGGAATACATCTACCTG-3′ R 5′-GAAGACGAATAGGTTTGAGTTC-3′	NM_013495
M_Lpl_1	*Lpl*	16956	Lipoprotein lipase	F 5′-GAGACTCAGAAAAAGGTCATC-3′ R 5′-GTCTTCAAAGAACTCAGATGC-3′	NM_008509
M_Pgc1a_1	*Pgc-1 α*	19017	Peroxisome proliferative activated receptor, gamma, coactivator 1 alpha	F 5′-TCCTCTTCAAGATCCTGTTAC-3′ R 5′-CACATACAAGGGAGAATTGC-3′	NM_008904
Antioxidant genes					
M_Cat_1	*Cat*	12359	Catalase	F 5′- CTCCATCAGGTTTCTTTCTTG-3′ R 5′- CAACAGGCAAGTTTTTGATG-3′	NM_009804
M_Gpx4_1	*Gpx4*	625249	Glutathione peroxidase 4	F 5′- TGGATAAGTACAGGGGTTTC-3′ R 5′- TAGCTGAGTGTAGTTTACGTC-3′	NM_008162
M_Gsr _1	*Gsr*	14782	Glutathione reductase	F 5′- GTTCACACAGGTTAAGGAAG-3′ R 5′- TATTCAGATTCAGGCCCTTAG-3′	X76341
Housekeeping gene					
M_Actb_1	*Actb*	11461	Actin beta	F 5′-GATGTATGAAGGCTTTGGTC-3′ R 5′-TGTGCACTTTTATTGGTCTC-3′	NM_007393
